# Supplementary material for: The Reissner Fiber in the Cerebrospinal Fluid Controls Morphogenesis of the Body Axis
Source: Curr Biol. 2018 Aug 6;28(15):2479–2486.e4. doi: 10.1016/j.cub.2018.05.079 (PMC6089837; doi:10.1016/j.cub.2018.05.079)
Supplement: Document S1. Figures S1–S4 and Table S1 [file mmc1.pdf]

Current Biology, Volume *28*

Supplemental Information

The Reissner Fiber in the Cerebrospinal Fluid

Controls Morphogenesis of the Body Axis

Yasmine Cantaut-Belarif, Jenna R. Sternberg, Olivier Thouvenin, Claire Wyart, and Pierre-Luc Bardet

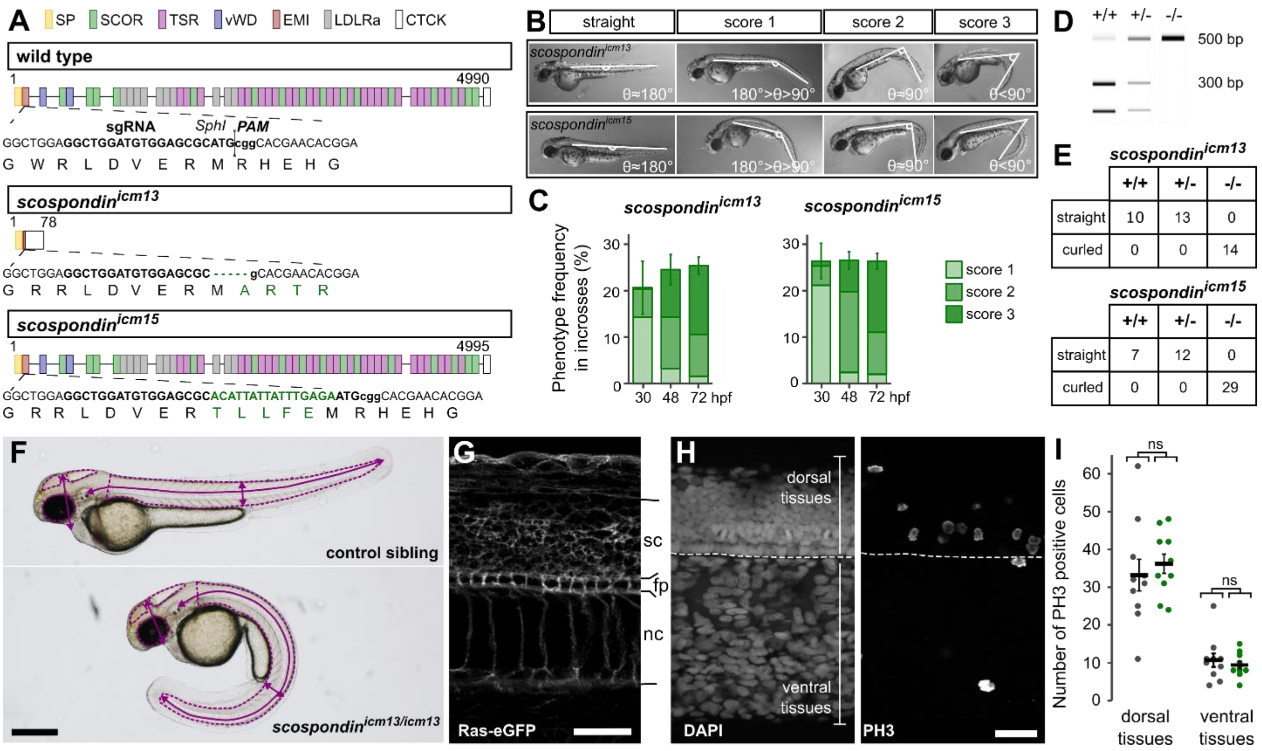

Figure S1. Embryonic posterior axis defects strictly correlate with *scospondin* mutated alleles, *related to Figure 1 and Table S1*

A. CRISPR/Cas9-mediated genome editing leading to a nonsense mutation, and a five amino acids insertion in the EMI domain of SCO-spondin for the *scospondin*^{icm13} and *scospondin*^{icm15} allele, respectively (SP: signal peptide, EMI: Emilin domain, SCOR: SCO-spondin repeats, vWD: von Willebrand D domain, LDLRa: Low-Density Lipoprotein Receptor type A Repeat, TSR: Thrombospondin type 1 Repeats, CTCK: C-terminal Cystine Knot, see [S1]). **B.** *scospondin* mutants show different levels of severity in body axis curvature at 48 hpf (score 1, 2, 3). Curvature defects are classified according to the angle (θ) between the tail and the rostral axis. **C.** Around 25% of embryos showed curvature defects from 30 hpf onwards, in agreement with Mendelian distributions. Curvature defects (%, mean ± SEM) were more pronounced over time for both *scospondin* alleles (from 30 to 72 hpf, n=386 and 248 embryos for *icm13* and *icm15* alleles respectively, n= 3 clutches). **D.** Embryos from *scospondin*$^{icm13/+}$ and *scospondin*$^{icm15/+}$ incrosses were genotyped at 72 hpf using the *Sph1* restriction site loss in *scospondin* mutants. **E.** All *scospondin* homozygous mutants showed the curled-down phenotype and it was observed neither in the *scospondin* heterozygous nor in wild type embryos. The same is true for trans-heterozygous *scospondin*$^{icm13/icm15}$ embryos (*data not shown*). **F.** To perform morphometric analysis of *scospondin* mutants, 48 hpf control and curled-down embryos from both alleles were measured for the eye, brain ventricles and tail area (dotted lines) as well as head and trunk height and tail length (solid lines). (Scale bar represents 1 mm.) **G.** To perform morphometric measurements at the level

of the trunk, 30 hpf control and *scospondin*$^{icm13/icm13}$ embryos injected at one cell stage with the Ras-eGFP mRNA were immunostained against GFP. The membrane-tag fluorescence was used to measure the spinal cord (sc), floor plate (fp) and notochord (nc) height in both straight and curled-down siblings. **H.** Representative images of a 30 hpf control embryo immunostained against phospho-histone 3 (PH3, right) allowing to detect cell proliferation. DAPI counterstaining of the nuclei (left) allowed differentiating dorsal and ventral tissues above and under the floorplate (dotted line) respectively. **I.** Quantification of PH3 positive cells in control (black) and *scospondin*$^{icm13/icm13}$ (green) embryos at 30 hpf in dorsal and ventral tissues. n=10 embryos for each condition. p= 0.57; 0.55, t= -0.58; 0.61 and df= 15; 14 for dorsal; ventral tissues respectively; two-tailed t-test. Scale bars represent 30 µm in **G** and **H**.

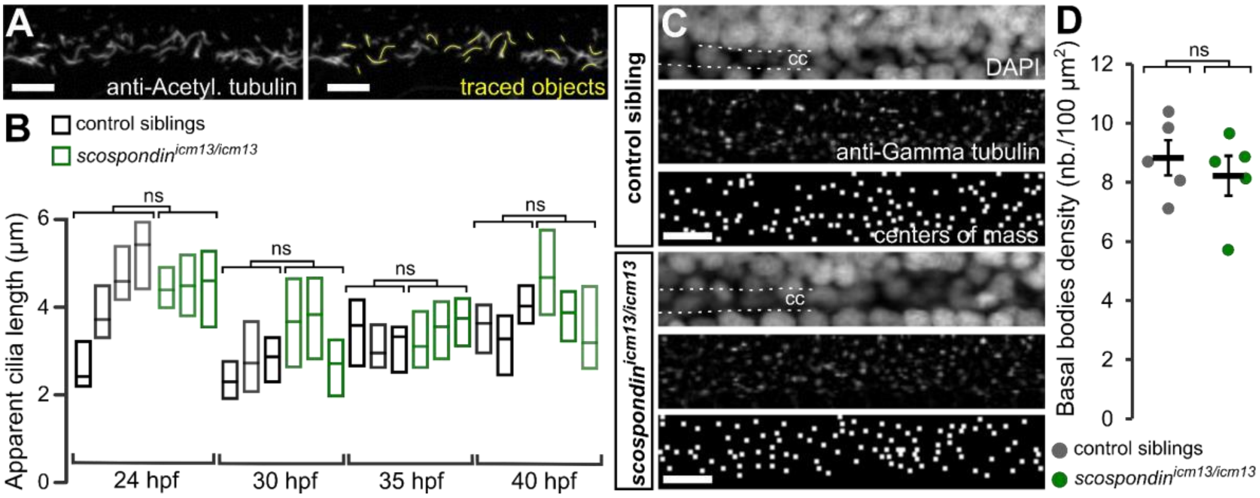

Figure S2. Cilia length and density are unchanged in *scospondin*$^{icm13/icm13}$ mutants, *related to Figure 2 and Video S1*

A. Z projection of a stack of lateral optical sections (depth= 3 µm) of the spinal cord of a 30 hpf embryo immunostained against Acetylated-tubulin (left) and after tracing to estimate cilia length (yellow). Scale bar represents 10 µm. **B**. Cilia length is similar in *scospondin*$^{icm13/icm13}$ embryos (green) compared to control siblings (black). (Median ± interquartile range, n= 83; 105; 95 and 95 and 67; 96; 94 and 89 cilia at 24; 30; 35 and 40 hpf for control and *scospondin*$^{icm13/icm13}$; p = 0.53; 0.14; 0.53 and 0.61, respectively. t= -0.7; -1.8; -0.67 and -0.55, respectively; df = 3.05; 3.4; 3.9 and 2.9, respectively, two-tailed t-test). Each box plot represents a single fish; color intensity reflects the number of measured objects for each fish. Similar results were obtained for *scospondin*$^{icm15/icm15}$ embryos (*data not shown*). **C, D.** Z projection of stacks of lateral optical sections (depth = 3 µm) of the spinal cord stained with DAPI and against Gamma-tubulin in a 30 hpf control sibling (top) and *scospondin*$^{icm13/icm13}$ curled-down embryo (bottom), allowing to detect basal bodies around the central canal (cc). Centers of mass of detected objects were used to quantify cilia density shown in **D** as the number of basal bodies per 100 µm^2. n= 5 control (black) and 5 *scospondin*$^{icm13/icm13}$ embryos (green). p= 0.52, t= 0.68, df= 8, two-tailed t-test. Each point corresponds to a single fish**.** Scale bars represent 10 µm in **C**.

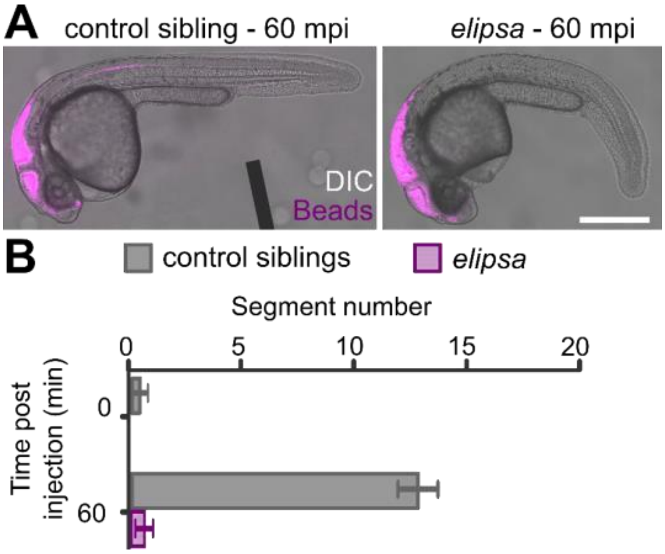

Figure S3. Transport of exogenous fluorescent beads in the cerebrospinal fluid is abolished in *elipsa* mutants with defective cilia, *related to Figure 3 and Video S2*.

A. Superimposed images of transmitted light (DIC) and fluorescent 20-nm diameter beads (magenta) injected in the third ventricle of 30 hpf control sibling and *elipsa* mutant embryos. Beads are transported down the central canal in straight control siblings, but not in *elipsa* mutants as shown 60 minutes post-injection (mpi). Scale bar represents 0.5 mm. The progression of the fluorescence front in the central canal is quantified in **B** as the segment number reached 60 minutes after injection (mean ± SEM) in control sibling (n = 3) and *elipsa* mutant embryos (n = 5) (p = 0.0014; t = 12.46, df = 2.84, two tailed t-test).

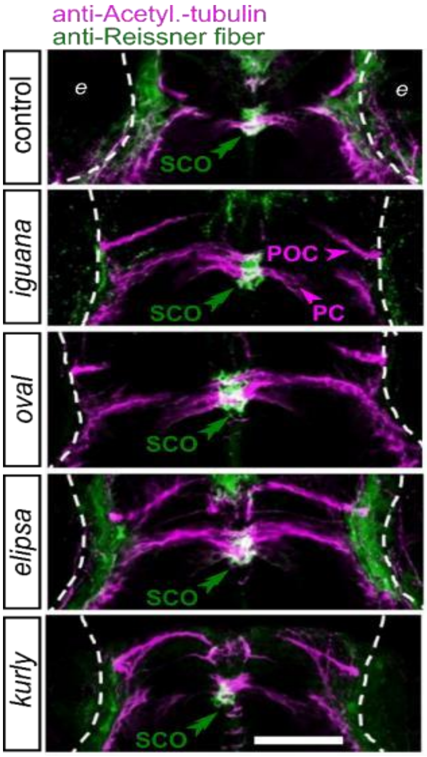

Figure S4. The sub-commissural organ is immunoreactive for the Reissner fiber material in mutants with defective cilia, *related to Figure 4*.

Z projection of stacks of dorsal optical sections (depth = 23 - 26 µm) of 48 hpf embryos immunostained against Acetylated-tubulin (magenta) allowing to detect axonal tracts (POC: post-optic commissure, PC: posterior commissure, arrowheads), and the Reissner fiber material (green) in the sub-commissural organ (SCO, double arrowheads). Control embryos (here an *iguana* sibling) as well as *iguana*, *oval*, *elipsa* and *kurly* mutants show immunoreactivity for the Reissner fiber material in the SCO. Rostral, top. e: eye. Scale bar represents 50 µm.

	allele	straight siblings	curled-down embryos	t-value	df	*p-value*
**eye area (µm^2)**	***icm13***	**55 368** +/- 2 532	**53 777** +/- 2 492	0.878	22.0	*0.390*
	icm15	**59 843** +/- 2 342	**56 828** +/- 2 432	1.751	20.8	*0.095*
**anterior ventral area (µm^2)**	***icm13***	**12 123** +/- 866	**12 859** +/- 1 023	-1.076	21.4	*0.294*
	icm15	**12 995** +/- 677	**12 216** +/- 715	1.551	20.8	*0.136*
**posterior ventral area (µm^2)**	***icm13***	**12 083** +/- 828	**14 009** +/- 2 090	-1.679	14.4	*0.115*
	icm15	**10 745** +/- 1085	**11 347** +/- 1 981	-0.522	15.6	*0.609*
**head height (µm)**	***icm13***	**449.9** +/- 10.0	**450.4** +/- 10.7	-0.062	21.9	*0.951*
	icm15	**452.3** +/- 8.6	**444.3** +/- 8.3	1.317	21.0	*0.202*
**tail area (µm^2)**	***icm13***	**427** +/- 21 **$.10^3$**	**409** +/- 14 **$.10^3$**	1.405	18.9	*0.176*
	icm15	**480** +/- 16 **$.10^3$**	**435** +/- 24 **$.10^3$**	3.045	17.3	*0.007*
**tail length (µm)**	***icm13***	**2 618** +/- 49	**2 695** +/- 45	-2.297	21.8	*0.032*
	icm15	**2 741** +/- 68	**2 660** +/- 206	0.727	12.2	*0.481*
**tail height (µm)**	***icm13***	**206.3** +/- 7.3	**196.0** +/- 4.4	2.373	18.1	*0.029*
	icm15	**217.0** +/- 6.3	**204.4** +/- 4.7	3.158	19.9	*0.009*
**spinal cord height (µm)**	***icm13***	**27.08** +/- 1.84	**26.39** +/- 3.73	0.329	5.8	*0.754*
**floor plate (µm)**	***icm13***	**3.79** +/- 0.55	**3.87** +/- 0.37	-0.242	7.0	*0.816*
**notochord (µm)**	***icm13***	**27.18** +/-3.23	**28.45** +/- 1.75	-0.679	6.2	*0.522*

Table S1. Morphometric analysis of *scospondin* mutant embryos, *related to Figure 1 and Figure S1*

We measured the size of different body parts of 48 hpf embryos, as depicted in Figure S1F. Between 5 and 6 embryos of each phenotype (curled-down mutants or straight siblings) from three independent clutches were measured for both alleles and displayed as means +/- confidence interval. We observed no signs of microcephaly (eye size and head height), hydrocephaly (head height, ventricle areas) or shortening of the body (tail length and area). Note that all p-values are above the significance threshold after Bonferroni correction (p< 0.007). We also performed measurement on confocal images of *scospondin*$^{icm13/icm13}$ and control siblings expressing a membrane-tethered GFP at 30 hpf (see Figure S1G). No major changes in the height of the spinal cord, the floor plate and the notochord were observed.

Supplemental References

S1. Meiniel, O., and Meiniel, A. (2007). The complex multidomain organization of SCO-spondin protein is highly conserved in mammals. Brain Res. Rev. 53, 321–327.
