# Supplementary material for: The Reissner Fiber in the Cerebrospinal Fluid Controls Morphogenesis of the Body Axis
Source: Curr Biol. 2018 Aug 6;28(15):2479–2486.e4. doi: 10.1016/j.cub.2018.05.079 (PMC6089837; doi:10.1016/j.cub.2018.05.079)
Supplement: Document S2. Article plus Supplemental Information [file mmc4.pdf]

Current Biology

# The Reissner Fiber in the Cerebrospinal Fluid Controls Morphogenesis of the Body Axis

## Graphical Abstract

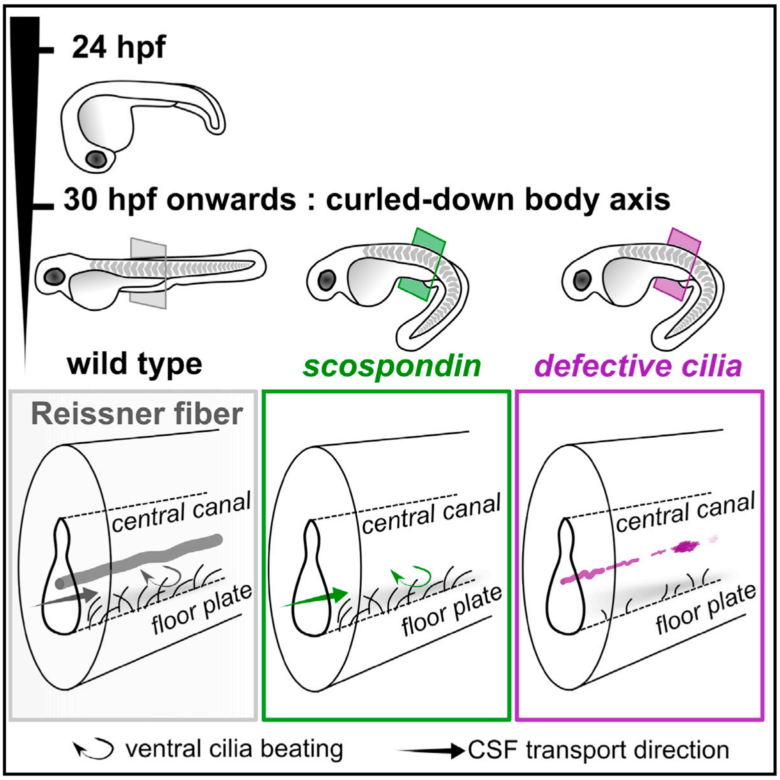


## Highlights

- Zebrafish *scospondin* mutants lose the Reissner fiber in the cerebrospinal fluid
- *scospondin* mutants show a curled-down axis similar to cilia-defective embryos
- *scospondin* mutants exhibit normal cilia and cerebrospinal fluid circulation
- Straight axis morphogenesis requires cilia-dependent assembly of the Reissner fiber


## Authors

Yasmine Cantaut-Belarif, Jenna R. Sternberg, Olivier Thouvenin, Claire Wyart, Pierre-Luc Bardet

## Correspondence

claire.wyart@icm-institute.org (C.W.), pierreluc.bardet@icm-institute.org (P.-L.B.)


## In Brief

Cantaut-Belarif et al. unravel a developmental role for the Reissner fiber, a long-known structure found in the cerebrospinal fluid. Zebrafish embryos lacking this extracellular thread develop a curled-down axis reminiscent of cilia-defective mutants. This fiber needs cilia to form and is required for straight axis morphogenesis.

Cantaut-Belarif et al., 2018, Current Biology *28*, 2479–2486
August 6, 2018 © 2018 The Authors. Published by Elsevier Ltd.
https://doi.org/10.1016/j.cub.2018.05.079

Current Biology
Report

CellPress

# The Reissner Fiber in the Cerebrospinal Fluid Controls Morphogenesis of the Body Axis

Yasmine Cantaut-Belarif,[1] Jenna R. Sternberg,[1,3] Olivier Thouvenin,[1,2] Claire Wyart,[1,4,5,*] and Pierre-Luc Bardet[1,4,*]
[1]Institut du Cerveau et de la Moelle Épinière (ICM), Inserm U 1127, CNRS UMR 7225, Sorbonne Université, 75013 Paris, France
[2]Institut Langevin ESPCI, PSL Research University, CNRS UMR 7587, 1 Rue Jussieu, 75005 Paris, France
[3]Present address: Department of Molecular and Cellular Biology, Harvard University, Cambridge, MA 02138, USA
[4]These authors contributed equally
[5]Lead Contact
*Correspondence: claire.wyart@icm-institute.org (C.W.), pierreluc.bardet@icm-institute.org (P.-L.B.)
https://doi.org/10.1016/j.cub.2018.05.079

## SUMMARY

**Organ development depends on the integration of coordinated long-range communication between cells. The cerebrospinal fluid composition and flow properties regulate several aspects of central nervous system development, including progenitor proliferation, neurogenesis, and migration [1–3]. One understudied component of the cerebrospinal fluid, described over a century ago in vertebrates, is the Reissner fiber. This extracellular thread forming early in development results from the assembly of the SCO-spondin protein in the third and fourth brain ventricles and central canal of the spinal cord [4]. Up to now, the function of the Reissner fiber has remained elusive, partly due to the lack of genetic invalidation models [4]. Here, by mutating the *scospondin* gene, we demonstrate that the Reissner fiber is critical for the morphogenesis of a straight posterior body axis. In zebrafish mutants where the Reissner fiber is lost, ciliogenesis and cerebrospinal fluid flow are intact but body axis morphogenesis is impaired. Our results also explain the frequently observed phenotype that mutant embryos with defective cilia exhibit defects in body axis curvature. Here, we reveal that these mutants systematically fail to assemble the Reissner fiber. We show that cilia promote the formation of the Reissner fiber and that the fiber is necessary for proper body axis morphogenesis. Our study sets the stage for future investigations of the mechanisms linking the Reissner fiber to the control of body axis curvature during vertebrate development.**

## RESULTS AND DISCUSSION

In most vertebrates, the Reissner fiber forms during development by the aggregation of a unique glycoprotein, SCO-spondin, initially secreted into the cerebrospinal fluid (CSF) by the floor plate (FP) and later only produced by the glandular cells of the sub-commissural organ (SCO) [4]. To monitor the Reissner fiber formation in zebrafish, we used an antibody raised against purified Reissner fiber fragments [5] to detect both secreting cells and the Reissner fiber in the CSF (Figures 1A, 1D, and 1E). As in other organisms, SCO-spondin is secreted in the zebrafish embryo by the SCO and the floor plate [6–8] (Figure 1A) to form the Reissner fiber. The structure extends from the ventricle along the full length of the central canal by 24 hr post-fertilization (hpf) (Figures 1A and 1D). The zebrafish SCO-spondin protein has a modular domain structure that is highly conserved in vertebrates [8, 9]. We generated *scospondin* mutants using CRISPR/Cas9-mediated genome editing by targeting the second coding exon (Figure S1A). We isolated the *scospondin*$^{icm13}$ allele with a frameshift mutation giving rise to a truncated protein devoid of any of the *scospondin* domains, likely to be null. A second, *scospondin*$^{icm15}$, allele exhibits five extra amino acids in the single EMI domain, a protein-protein interaction domain found in the Emilin protein family (Figure S1A) [8, 10]. For both alleles, incross of the heterozygous carrier led to 25% of embryos with a posterior curled-down body axis (Figures 1B and 1C), a phenotype previously observed but unexplained in mutants with defective cilia [11–15]. The abnormal curvature observed from 30 hpf onward gradually increased over time (Figures S1B and S1C). The curled-down phenotype was only observed in *scospondin* homozygous mutants (Figures S1D and S1E). Therefore, it was used for the rest of the study to identify homozygous mutant embryos after 30 hpf. In *scospondin* mutants, we did not observe other phenotypes associated with cilia dysfunction, such as kidney cysts or hydrocephalus [11, 16], or other gross morphological or proliferation defects (Figures S1F–S1I; Table S1; data not shown).

In all mutant embryos for both *scospondin* alleles, we observed as early as 24 hpf (i.e., 6 hr prior to the emergence of the curled-down phenotype) the lack of the Reissner fiber in the third brain ventricle and in the central canal (Figure 1D). Although the Reissner material was absent from the secretory structures of the *scospondin*$^{icm13/icm13}$ mutants, *scospondin*$^{icm15/icm15}$ embryos exhibited immunoreactivity for the Reissner material in the SCO and floor plate (Figure 1E). Because the signal peptide is unaffected by the insertion, this observation suggests that the abnormal protein is still secreted into the CSF but fails to form a fiber. Altogether, these data demonstrate that compromising the assembly of SCO-spondin into the Reissner fiber in the CSF disrupts the curvature of the posterior axis during embryogenesis.


This is an open access article under the CC BY license (http://creativecommons.org/licenses/by/4.0/).

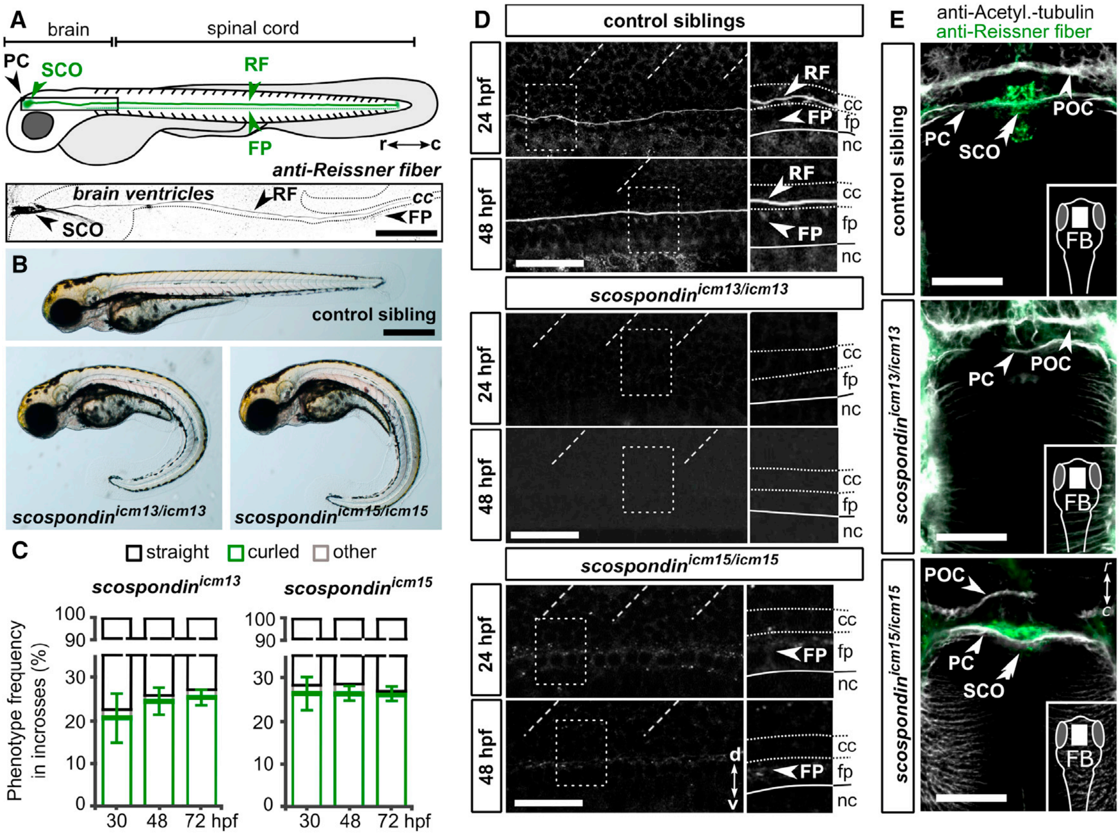


**Figure 1. Mutations in *scospondin* Lead to the Absence of the Reissner Fiber and Defects in Body Axis Formation**

(A) The Reissner fiber (RF) is localized in posterior ventricles of the brain and spinal central canal. At embryonic stages, SCO-spondin is secreted in the cerebrospinal fluid from the sub-commissural organ (SCO) below the posterior commissure (PC) and from the floor plate (FP) to form the fiber. Top: a scheme based on several immunohistochemistry experiments (below). Bottom: Z projection of a stack of a few lateral optical sections of the brain ventricles and rostral central canal (cc) of a 72 hpf embryo immunostained against the Reissner fiber (arrow). c, caudal; r, rostral. Scale bar represents 100 μm.

(B) 72 hpf *scospondin*$^{icm13/icm13}$ and *scospondin*$^{icm15/icm15}$ larvae showing curled-down phenotypes. Scale bar represents 500 μm.

(C) Proportion of curled-down phenotype over developmental time in both *scospondin* allele incrosses (mean ± SEM; n = 386 and 248 embryos for *scospondin*$^{icm13/icm13}$ and *scospondin*$^{icm15/icm15}$, respectively, from three independent clutches). The abnormal curvature of the body axis is detected from 30 hpf onward. Gastrulation-defective embryos were negligible ("other," gray).

(D) Z projection of a stack of lateral optical sections (depth 4–5 μm) of the spinal cord immunostained against the Reissner fiber in control and *scospondin* mutants at 24 hpf (top) and 48 hpf (bottom). Both mutants are deprived of the Reissner fiber in the central canal from 24 hpf onward but immunoreactivity is detected in the floor plate of *scospondin*$^{icm15/icm15}$ (see insets on right panels highlighting the dotted-box regions) (n = 33; 63 control embryos, n = 7; 19 *scospondin*$^{icm15/icm15}$ embryos, n = 12; 33 *scospondin*$^{icm13/icm13}$ at 24; 48 hpf, respectively). d, dorsal; nc, notochord; v, ventral. Scale bars represent 40 μm.

(E) Z projection of stacks of dorsal optical sections (depth 23–26 μm) of 48 hpf forebrains (FBs) immunostained for acetylated tubulin (gray) and the Reissner fiber (green) show Reissner fiber material in SCO (double arrowheads) of control and *scospondin*$^{icm15/icm15}$ embryos but not *scospondin*$^{icm13/icm13}$. Arrows indicate axonal commissures. POC, post-optic commissure. Scale bars represent 30 μm.

See also Figure S1 and Table S1.

It has long been observed that defective structure or motility of cilia leads to a curled-down posterior body axis in zebrafish embryos [11–15]. However, the mechanisms leading to this early defect have not been explained to date, and it is unclear whether this phenotype arises from ciliary defects in the central nervous system or in other tissues. Due to the conspicuous similarity between the curled-down phenotypes of *scospondin* mutants and previously described mutations affecting ciliogenesis and/or ciliary functions, we asked whether the Reissner fiber was necessary for ciliary functions. We investigated the formation and maintenance of cilia projecting into the lumen of the central canal of the spinal cord where the Reissner fiber forms. We first verified that mutant embryos for the *traf3ip1* gene encoding an essential protein for early ciliogenesis (*traf3ip1*$^{tp49d}$ mutant, further referred to as *elipsa* [13]) exhibited an obvious decrease in density and apparent length of cilia projecting into the CSF at 30 hpf (Figure 2A). In contrast, *scospondin*$^{icm13/icm13}$ embryos showed similar cilia length (Figures 2A, S2A, and S2B) and cilia density (Figures S2C and S2D) compared to control siblings. Second, we investigated the orientation and beating of the ventral motile cilia of the floor plate cells that are tilted in the posterior direction [17]. In 30 hpf *Tg(β-actin:Arl13b-GFP; scospondin*$^{icm13/icm13}$*)* mutant embryos, motile cilia inserted into the ventral central canal showed no difference in orientation compared to wild-type siblings (Figure 2B; Video S1). Altogether, we found no obvious ciliary defects in *scospondin* mutants,

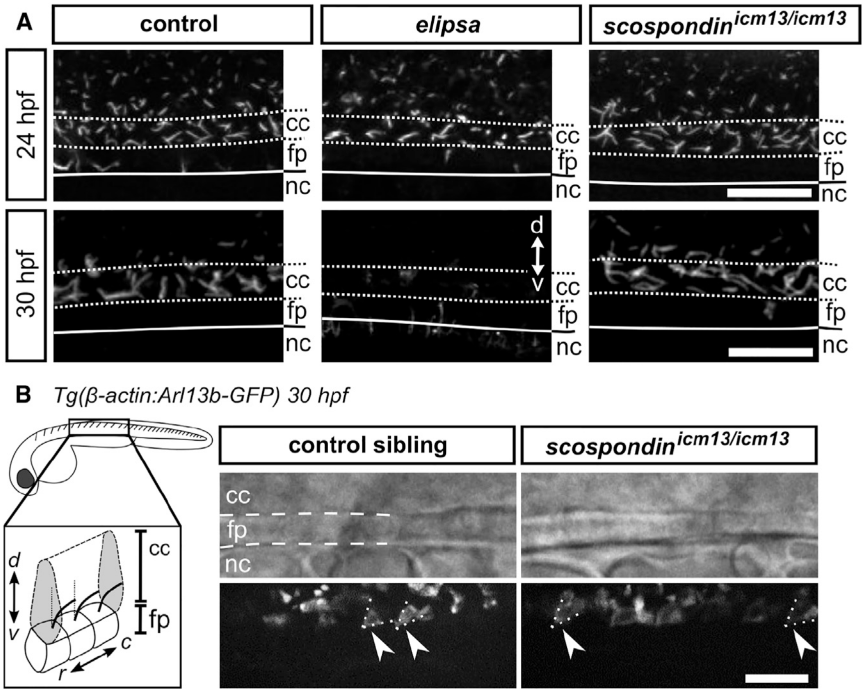


**Figure 2. Structural and Dynamic Properties of Cilia Appear Intact in the *scospondin*$^{icm13/icm13}$ Mutant**

(A) Z projection of stacks of lateral optical sections (depth 4–5 μm) of spinal cord immunostained against acetylated tubulin show intact cilia projecting into the central canal of control and *scospondin*$^{icm13/icm13}$ larvae at 24 (top) and 30 hpf (bottom). In comparison, *elipsa* embryos exhibit fewer cilia at 24 hpf, which are not maintained at 30 hpf. Scale bars represent 15 μm.

(B) Time projection from a 30-s-long time series acquired at 17 Hz and indicating movement of cilia expressing GFP in *Tg(β-actin:Arl13b-GFP; scospondin*$^{icm13/icm13}$*)* animals (right) and control siblings (left). Note the similarity in position (posterior tilt, dashed lines) and beating amplitude of motile cilia in the central canal (arrowheads) in mutant embryos compared to control siblings. The schematic summarizes our observations. Rostral, left; dorsal, top. Scale bar represents 10 μm.

See also Figure S2 and Video S1.

neither in length and density nor in motility, suggesting that formation, maintenance, beating, and polarization of cilia do not require the formation of the Reissner fiber in the central canal.

Measurements of CSF flow in the brain ventricles reveal complex CSF dynamics that correlate with the orientation of ependymal motile cilia, suggesting that the directional transport of CSF signaling molecules may be driven by coordinated cilia beating patterns [18]. Recently, the late induction of defects in cilia motility and polarization has been associated with torsion of the spine in juvenile zebrafish, reminiscent of human adolescent idiopathic scoliosis [19]. This observation led to the hypothesis that CSF circulation may contribute to body axis maintenance in juveniles. One could expect a similar link at embryonic stages, where the curled-down phenotype in the absence of the Reissner fiber could be due to a reduction in CSF flow. We therefore tested whether the Reissner fiber contributes to CSF flow and transport, by injecting exogenous fluorescent beads into the hindbrain ventricle at 30 hpf (Figure 3). As detailed for wild-type embryos in another manuscript (J.R.S., A.E. Prendergast, L. Brosse, Y.C.-B., O.T., A. Orts-Del'Immagine, L. Castillo, L. Djenoune, S. Kurisu, J.R. McDearmid, P.-L.B., C. Boccara, H. Okamoto, P. Delmas, and C.W., unpublished data), we observed a bidirectional CSF flow in the central canal of the spinal cord. Whereas injected beads flowed rostral to caudal on the ventral side of the central canal, they circulated in the opposite direction on the dorsal side (Figures 3A–3C; Video S2). When we conducted the same approach on *scospondin*$^{icm13/icm13}$ mutants, we observed that the CSF flow was bidirectional as well, with similar average particle velocities on the ventral and dorsal side (Figure 3C; Video S2).

To estimate the effective rostro-caudal transport associated with CSF flow, we then measured the progression of the fluorescent bead front through the central canal at 30 hpf (Figure 3D). In *elipsa* mutants, which display early ciliogenesis defects, transport down the central canal was compromised (Figure S3; as previously shown in *ift88*$^{tz288b}$*/oval* mutants [11]). In contrast, transport was unaffected in *scospondin*$^{icm13/icm13}$ mutants compared to control siblings (Figure 3E). Indirect evaluation of CSF transport in adult rat spinal cord after immunosuppression of the Reissner fiber previously showed a reduction in transport [20], suggesting that the Reissner fiber was necessary for CSF flow. Our direct measurements of CSF flow with fluorescent beads rule this hypothesis out in *scospondin* mutant zebrafish embryos, as we observed no change in bead velocity and no difference in net flow. Our results therefore demonstrate that abnormal posterior axis curvature during embryogenesis in *scospondin* mutants does not result from the abolition of the CSF circulation in the central canal.

Because the lack of the Reissner fiber in *scospondin* mutants does not affect ciliary structure and motility, nor the net CSF transport in the central canal, we investigated whether ciliary function was required for the proper assembly of the fiber. Because several genes encoding proteins essential for cilia function have cilia-independent roles [17, 21], we chose to study four independent mutants showing defects in cilia maintenance, motility, and/or polarity: *elipsa* and *oval* mutants, the ciliogenesis-defective mutant *dzip1*$^{ts294e}$ (hereafter referred to as *iguana* [14]), and the *cfap298*$^{tm304}$ mutant (hereafter referred to as *kurly*), where cilia motility and polarity are disrupted [22]. In all four mutants, the structures responsible for SCO-spondin secretion were still immunoreactive (Figure S4) but the Reissner fiber was drastically compromised at 48 hpf in the central canal (Figures 4A–4C). Instead of a fiber, we detected improper assemblies of material that differed along the rostro-caudal axis for each mutant as well as in-between mutants (Figure 4C). These differences might be explained by a difference in

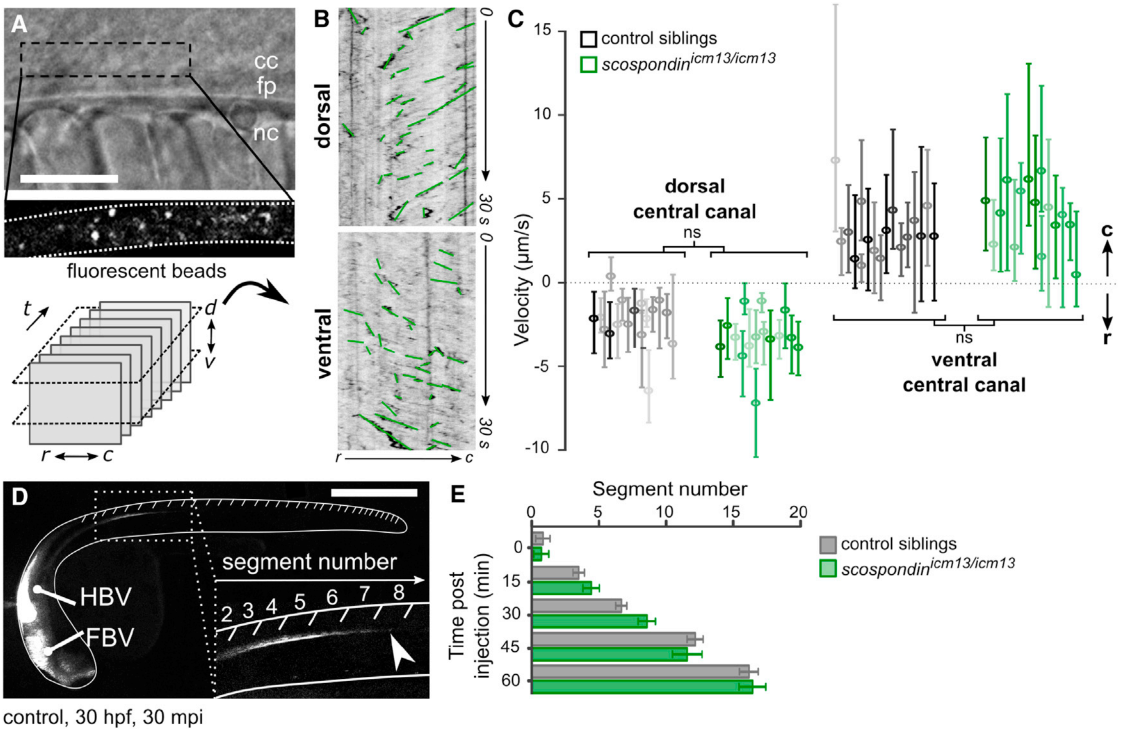


**Figure 3. Cerebrospinal Fluid Properly Flows in the Central Canal of the *scospondin*$^{icm13/icm13}$ Mutant**

(A and B) Lateral view of the central canal (A; transmitted, top) filled with fluorescent beads (bottom). Scale bar represents 30 μm. Time-lapse images at two positions (dorsal and ventral) are represented with the rostro-caudal axis as the horizontal axis and time as the vertical one (B). Kymographs reveal a bidirectional flow, with bead trajectories pointing at opposite directions in the dorsal and ventral central canal. Bead trajectories (green) were used to estimate bead velocities along the rostro-caudal axis.

(C) Bead velocities were similar in control siblings (black) and *scospondin*$^{icm13/icm13}$ (green) embryos in the dorsal and ventral central canal (control: n = 570; 584; mutants: n = 582; 634 trajectories in the dorsal; ventral position, respectively; p = 0.07 and 0.33, t = −1.8 and 0.99, and degrees of freedom [df] = 28.8 and 16 in the dorsal and ventral position, respectively, two-tailed t test). Values are given as median ± interquartile range; one boxplot per fish; color intensity reflects the number of measured trajectories per fish. ns, not significant.

(D) 30 hpf zebrafish embryo injected with fluorescent beads in brain ventricles shows transport down the central canal (inset) 30 min post-injection (mpi), reaching here the 8th somite (arrowhead). FBV, forebrain ventricle; HBV, hindbrain ventricle. Scale bar represents 0.5 mm.

(E) The fluorescence front moving down the central canal over time was indistinguishable in control siblings (gray) and *scospondin*$^{icm13/icm13}$ mutants (green). As a consequence, the fluorescence reached the same level 1 hr after injection (n = 6 versus 7, respectively, p = 0.83, t = −0.21, df = 10.3, two-tailed t test). Error bars are mean ± SEM.

See also Figure S3 and Video S2.

penetrance of the mutations, possibly due to variation in the maternal contribution. Although a continuous Reissner fiber was detected in the rostral central canal in both *iguana* and *oval* mutants (Figure 4C), we only observed short and disjointed fiber pieces in the caudal central canal. *elipsa* mutants were also characterized by the presence of aggregated forms of Reissner material, whereas *kurly* mutants displayed only diffuse states of Reissner material in the CSF (Figure 4C). Altogether, our data indicate that cilia are critical for the formation of the Reissner fiber.

To test whether defects in the Reissner fiber formation preceded the curled-down phenotype, we immunostained *elipsa* and *kurly* mutant embryos for the Reissner material at 24 hpf, shortly before the onset of body curvature defects (Figures 4D and 4E). Although we could detect Reissner material in the central canal at this early stage, we only observed improper aggregates (Figures 4D and 4E) resembling the phenotype of the *oval* mutants at 48 hpf. This observation indicates that the Reissner fiber disorganization precedes the appearance of the curled-down phenotype in mutants with defective cilia. Altogether, our results show that cilia function is required for the assembly of the Reissner fiber in the central canal during embryogenesis, thereby controlling morphogenesis of a straight posterior body axis.

Because the *kurly* mutant does not affect ciliogenesis but only motility and polarity [22], our results suggest that cilia motility and polarity are in themselves crucial for the correct formation of the Reissner fiber. This process may relate to *in vitro* studies where subjecting proteins to a defined flow field promotes their aggregation [23], suggesting that protein aggregation can be sensitive to hydrodynamic flow changes. Although we cannot exclude other explanations, such as defects in secretion or post-translational modifications in mutants with defective cilia, our observations in the *kurly* mutant suggest that cilia-driven flow enables the proper aggregation of the fibrous SCO-spondin protein into a mature Reissner fiber.

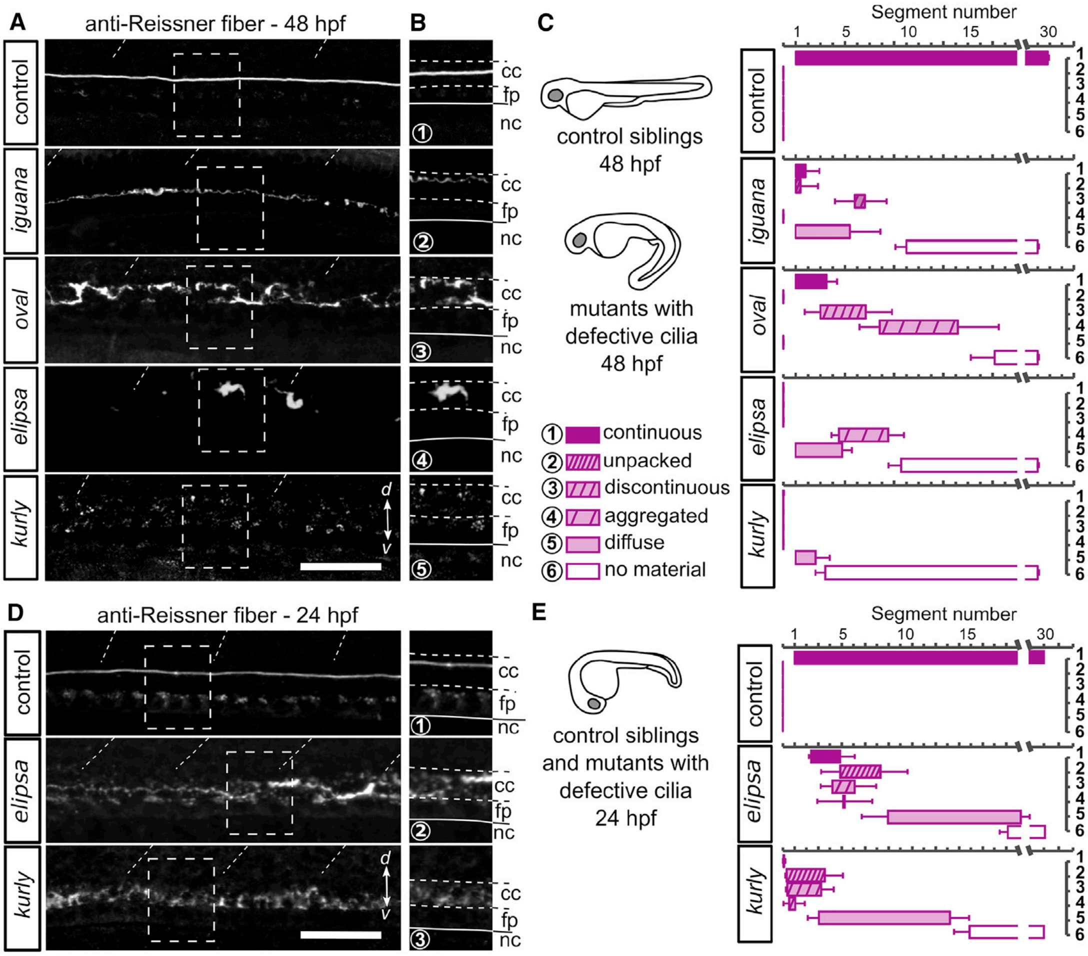


**Figure 4. Intact Cilia Are Necessary for the Formation of the Reissner Fiber in the Central Canal**

(A) Z projection of stacks of lateral optical sections (depth 4–5 μm) of spinal cord at 48 hpf showing modifications of immunoreactivity for the Reissner fiber in mutants with defective cilia (*iguana*, *oval*, *elipsa*, and *kurly*) compared to control siblings, where a continuous fiber runs along the entire central canal. d, dorsal; v, ventral. Scale bar represents 30 μm.

(B) Zoom of regions boxed in (A) showing depositions of the Reissner material in the central canal. Deposits occur as continuous densely packed fiber (1), continuous unpacked fiber (2), discontinuous loosely packed material (3), aggregated material (4), diffuse material (5), and absence of material (6). cc, central canal; fp, floor plate; nc, notochord.

(C) Distribution of defects in the Reissner material along the rostro-caudal axis and for each mutant displayed as mean segment number ± SEM (n = 24; 11; 6; 8; 5 embryos for control siblings; *iguana*; *oval*; *elipsa*, and *kurly*, respectively).

(D and E) Before *elipsa* and *kurly* embryos develop the curled-down phenotype (24 hpf embryo), defects in the Reissner material can be observed in the central canal. Right panels (D) show zoom from regions boxed in left panels with depositions of the Reissner material in the central canal; the numbers correspond to the equivalent defects at 48 hpf (depicted in B and C). Distribution of defects in the Reissner material along the rostro-caudal axis for the two mutants at 24 hpf (E) (bottom, n = 12 and 19 embryos for *elipsa* and *kurly*, respectively) compared to control siblings (top; n = 28). Scale bar represents 30 μm. Cc, central canal; fp, floor plate; nc, notochord.

See also Figure S4.

To our knowledge, we achieved the first targeted and permanent loss of function of the *scospondin* gene. The curled-down phenotype of homozygous *scospondin* mutants is highly reproducible and fully penetrant. No other morphological defects, in particular associated with ciliary dysfunctions such as hydrocephalus, were observed (Figures S1F–S1I; Table S1). The *scospondin* mutant larvae eventually die around 10 days post-fertilization, probably as a consequence of their inability to inflate their swim bladder and feed. This is in contrast with a study in chicken embryos, where transient *scospondin* loss of function by short hairpin RNA (shRNA) electroporation led to severe defects in neurogenesis giving rise to a brain malformation, presumably due to the secreted SCO-spondin independent of fiber formation [24]. Although our *scospondin*$^{icm13}$ allele is predicted to be a null that would supposedly abolish all form of SCO-spondin secretion, we failed to detect any major CNS malformation or proliferation defects (Figures S1G–S1I; Table S1). These distinct phenotypes may originate from differences between species or a possible technical caveat of transient electroporation in chick.

Surprisingly, our results show that the mere insertion of five extra amino acids into the EMI domain of the *scospondin*$^{icm15}$ allele prevents Reissner fiber formation and recapitulates the phenotype resulting from the truncation of the whole protein in *scospondin*$^{icm13}$. This observation suggests that the EMI domain, also found in the multimodular organization of other extracellular matrix proteins such as Emilins [10, 25], is functionally important for the Reissner fiber formation through protein-protein interactions that remain to be identified.

Remarkably, the only common feature we observed in the six mutants for *scospondin* or ciliary genes with a curled-down phenotype is the absence of a continuous Reissner fiber. Our results indicate that the Reissner fiber, not CSF flow per se, is necessary for the morphogenesis of the posterior axis during embryogenesis. This conclusion links the composition of the CSF to the shape of the embryonic posterior axis, a link that was difficult to predict from mutants with defective cilia affecting virtually all embryonic tissue. Considering the very small diameter of the Reissner fiber in comparison to the rest of the posterior body, and the intense mechanical forces generated by the twitching muscles and the notochord at embryonic stages, it is unlikely that the fiber could control morphogenesis by directly stiffening the posterior body axis. The action of the Reissner fiber most likely involves downstream signals that remain to be determined. Our discovery of a critical role of the Reissner fiber in body morphogenesis provides a mechanistic explanation for the unresolved observation that CSF-drained zebrafish embryos develop a mild curled-down posterior axis [26]. Thus, the investigation of the role of the Reissner fiber opens an alternative avenue to re-evaluating recent reports associating changes in CSF flow and defects of spine organogenesis in juveniles [27].

Since the discovery of the Reissner fiber and the SCO, various hypotheses have been proposed for their functions, including the regulation of hydromineral balance, CSF production, composition, circulation, or detoxification [28]. Early work also suggested that these structures could control the morphogenesis of the tail in juvenile amphibians [29, 30], although this hypothesis relied on surgical procedures with little precision and is therefore hard to interpret. Interestingly, natural lordotic specimens of *Sparus aurata* showed important alterations in Reissner fiber condensation in the central canal of the spinal cord [31]. Our results based on replicable genetic ablations of five different genes all converge to demonstrate the role of the Reissner fiber in body morphogenesis. A century and a half after its discovery, this study provides new evidence for a critical role of the Reissner fiber during development. Now, our study opens a new field of investigation centered on bioactive molecules in the CSF of the ventricular space that could interact with the binding motifs of the SCO-spondin for monoamines [32] and low-density lipoproteins [33], and thereby regulate early body axis morphogenesis in vertebrates.

## STAR★METHODS

Detailed methods are provided in the online version of this paper and include the following:

- KEY RESOURCES TABLE
- CONTACT FOR REAGENT AND RESOURCE SHARING
- EXPERIMENTAL MODEL AND SUBJECT DETAILS
  - Zebrafish
- METHOD DETAILS
  - scospondin mutants generation and genotyping
  - scospondin mutants phenotype scoring
  - Immunohistochemistry and fixed tissue imaging
  - Quantification of apparent cilia length and density
  - Live imaging of spinal cord cilia
  - Fluorescent beads injection in the CSF
  - Beads transport analysis
  - Fluorescent beads tracking
  - Analysis of Reissner fiber formation defects
- QUANTIFICATION AND STATISTICAL ANALYSIS

## SUPPLEMENTAL INFORMATION

Supplemental Information includes four figures, one table, and two videos and can be found with this article online at https://doi.org/10.1016/j.cub.2018.05.079.

## ACKNOWLEDGMENTS

We thank Prof. Brian Ciruna for the *Tg(β-actin:Arl13b-GFP)* line, Prof. Rebecca Burdine for the *kurly* (*tm304* allele) mutant, Prof. Jarema Malicki for the *elipsa* mutant (*ift54/traf3ip*), Prof. Sylvie Schneider-Maunoury and Dr. Christine Vesque for providing us the Ras-eGFP plasmid, Dr. Stéphane Gobron for the antibody directed against the Reissner fiber, the ICM.Quant imaging facility for instrument use and scientific and technical assistance, François-Xavier Lejeune and Ivan Moszer for statistical analysis, and Sophie Nunes-Figueiredo, Natalia Maties, Bogdan Buzurin, and Monica Dicu for fish care. We thank for critical feedback Drs. Christine Vesque, Sylvie Schneider-Maunoury, Nicolas Renier, and Bassem Hassan and all members of the Wyart lab (https://wyartlab.org/). This work was supported by ERC Starting Grant "Optoloco" (grant no. 311673), Human Frontier Science Program (HFSP) Research Grant (grant no. RGP063), the New York Stem Cell Foundation (NYSCF) (grant no. NYSCF-R-NI39), and the Michelin Corporate Foundation (grant no. 087). The research leading to these results has received funding from the program "Investissements d'avenir" ANR-10-IAIHU-06 (Big Brain Theory ICM Program and postdoctoral fellowship program for O.T.) and ANR-11-INBS-0011 (NeurATRIS: Translational Research Infrastructure for Biotherapies in Neurosciences).

## AUTHOR CONTRIBUTIONS

Y.C.-B., C.W., and P.-L.B. designed the experiments and wrote the manuscript. P.-L.B. generated the mutant alleles. P.-L. B. and Y. C.-B. quantified changes in body morphogenesis. Y.C.-B. performed and analyzed experiments requiring immunohistochemistry, analysis of cilia length, and CSF flow measurements and analysis. C.W., J.R.S., and O.T. contributed to CSF flow experiment optimization and particle velocity estimates from kymographs. C.W. and P.-L.B. conceived and supervised the research.

## DECLARATION OF INTERESTS

The authors declare no competing interests.

Received: March 20, 2018
Revised: May 15, 2018
Accepted: May 25, 2018
Published: July 26, 2018

## REFERENCES

1. Lehtinen, M.K., Zappaterra, M.W., Chen, X., Yang, Y.J., Hill, A.D., Lun, M., Maynard, T., Gonzalez, D., Kim, S., Ye, P., et al. (2011). The cerebrospinal

fluid provides a proliferative niche for neural progenitor cells. Neuron *69*, 893–905.

2. Paul, A., Chaker, Z., and Doetsch, F. (2017). Hypothalamic regulation of regionally distinct adult neural stem cells and neurogenesis. Science *356*, 1383–1386.
3. Sawamoto, K., Wichterle, H., Gonzalez-Perez, O., Cholfin, J.A., Yamada, M., Spassky, N., Murcia, N.S., Garcia-Verdugo, J.M., Marin, O., Rubenstein, J.L., et al. (2006). New neurons follow the flow of cerebrospinal fluid in the adult brain. Science *311*, 629–632.
4. Rodríguez, E.M., Rodríguez, S., and Hein, S. (1998). The subcommissural organ. Microsc. Res. Tech. *41*, 98–123.
5. Didier, R., Dastugue, B., and Meiniel, A. (1995). The secretory material of the subcommissural organ of the chick embryo. Characterization of a specific polypeptide by two-dimensional electrophoresis. Int. J. Dev. Biol. *39*, 493–499.
6. Lichtenfeld, J., Viehweg, J., Schützenmeister, J., and Naumann, W.W. (1999). Reissner's substance expressed as a transient pattern in vertebrate floor plate. Anat. Embryol. (Berl.) *200*, 161–174.
7. Lehmann, C., and Naumann, W.W. (2005). Axon pathfinding and the floor plate factor Reissner's substance in wildtype, cyclops and one-eyed pinhead mutants of *Danio rerio*. Brain Res. Dev. Brain Res. *154*, 1–14.
8. Meiniel, O., Meiniel, R., Lalloué, F., Didier, R., Jauberteau, M.O., Meiniel, A., and Petit, D. (2008). The lengthening of a giant protein: when, how, and why? J. Mol. Evol. *66*, 1–10.
9. Meiniel, O., and Meiniel, A. (2007). The complex multidomain organization of SCO-spondin protein is highly conserved in mammals. Brain Res. Brain Res. Rev. *53*, 321–327.
10. Doliana, R., Bot, S., Bonaldo, P., and Colombatti, A. (2000). EMI, a novel cysteine-rich domain of EMILINs and other extracellular proteins, interacts with the gC1q domains and participates in multimerization. FEBS Lett. *484*, 164–168.
11. Kramer-Zucker, A.G., Olale, F., Haycraft, C.J., Yoder, B.K., Schier, A.F., and Drummond, I.A. (2005). Cilia-driven fluid flow in the zebrafish pronephros, brain and Kupffer's vesicle is required for normal organogenesis. Development *132*, 1907–1921.
12. Tsujikawa, M., and Malicki, J. (2004). Intraflagellar transport genes are essential for differentiation and survival of vertebrate sensory neurons. Neuron *42*, 703–716.
13. Omori, Y., Zhao, C., Saras, A., Mukhopadhyay, S., Kim, W., Furukawa, T., Sengupta, P., Veraksa, A., and Malicki, J. (2008). Elipsa is an early determinant of ciliogenesis that links the IFT particle to membrane-associated small GTPase Rab8. Nat. Cell Biol. *10*, 437–444.
14. Brand, M., Heisenberg, C.P., Warga, R.M., Pelegri, F., Karlstrom, R.O., Beuchle, D., Picker, A., Jiang, Y.J., Furutani-Seiki, M., van Eeden, F.J., et al. (1996). Mutations affecting development of the midline and general body shape during zebrafish embryogenesis. Development *123*, 129–142.
15. Karlstrom, R.O., Trowe, T., Klostermann, S., Baier, H., Brand, M., Crawford, A.D., Grunewald, B., Haffter, P., Hoffmann, H., Meyer, S.U., et al. (1996). Zebrafish mutations affecting retinotectal axon pathfinding. Development *123*, 427–438.
16. Sullivan-Brown, J., Schottenfeld, J., Okabe, N., Hostetter, C.L., Serluca, F.C., Thiberge, S.Y., and Burdine, R.D. (2008). Zebrafish mutations affecting cilia motility share similar cystic phenotypes and suggest a mechanism of cyst formation that differs from pkd2 morphants. Dev. Biol. *314*, 261–275.
17. Borovina, A., Superina, S., Voskas, D., and Ciruna, B. (2010). Vangl2 directs the posterior tilting and asymmetric localization of motile primary cilia. Nat. Cell Biol. *12*, 407–412.
18. Faubel, R., Westendorf, C., Bodenschatz, E., and Eichele, G. (2016). Cilia-based flow network in the brain ventricles. Science *353*, 176–178.
19. Grimes, D.T., Boswell, C.W., Morante, N.F.C., Henkelman, R.M., Burdine, R.D., and Ciruna, B. (2016). Zebrafish models of idiopathic scoliosis link cerebrospinal fluid flow defects to spine curvature. Science *352*, 1341–1344.
20. Cifuentes, M., Rodríguez, S., Pérez, J., Grondona, J.M., Rodríguez, E.M., and Fernández-Llebrez, P. (1994). Decreased cerebrospinal fluid flow through the central canal of the spinal cord of rats immunologically deprived of Reissner's fibre. Exp. Brain Res. *98*, 431–440.
21. Bizet, A.A., Becker-Heck, A., Ryan, R., Weber, K., Filhol, E., Krug, P., Halbritter, J., Delous, M., Lasbennes, M.C., Linghu, B., et al. (2015). Mutations in TRAF3IP1/IFT54 reveal a new role for IFT proteins in microtubule stabilization. Nat. Commun. *6*, 8666.
22. Jaffe, K.M., Grimes, D.T., Schottenfeld-Roames, J., Werner, M.E., Ku, T.S.J., Kim, S.K., Pelliccia, J.L., Morante, N.F.C., Mitchell, B.J., and Burdine, R.D. (2016). c21orf59/kurly controls both cilia motility and polarization. Cell Rep. *14*, 1841–1849.
23. Dobson, J., Kumar, A., Willis, L.F., Tuma, R., Higazi, D.R., Turner, R., Lowe, D.C., Ashcroft, A.E., Radford, S.E., Kapur, N., and Brockwell, D.J. (2017). Inducing protein aggregation by extensional flow. Proc. Natl. Acad. Sci. USA *114*, 4673–4678.
24. Vera, A., Stanic, K., Montecinos, H., Torrejón, M., Marcellini, S., and Caprile, T. (2013). SCO-spondin from embryonic cerebrospinal fluid is required for neurogenesis during early brain development. Front. Cell. Neurosci. *7*, 80.
25. Callebaut, I., Mignotte, V., Souchet, M., and Mornon, J.P. (2003). EMI domains are widespread and reveal the probable orthologs of the *Caenorhabditis elegans* CED-1 protein. Biochem. Biophys. Res. Commun. *300*, 619–623.
26. Chang, J.T., Lehtinen, M.K., and Sive, H. (2016). Zebrafish cerebrospinal fluid mediates cell survival through a retinoid signaling pathway. Dev. Neurobiol. *76*, 75–92.
27. Boswell, C.W., and Ciruna, B. (2017). Understanding idiopathic scoliosis: a new zebrafish school of thought. Trends Genet. *33*, 183–196.
28. Grondona, J.M., Hoyo-Becerra, C., Visser, R., Fernández-Llebrez, P., and López-Ávalos, M.D. (2012). The subcommissural organ and the development of the posterior commissure. Int. Rev. Cell Mol. Biol. *296*, 63–137.
29. Rühle, H.-J. (1971). Anomalien im Wachstum der Achsenorgane nach experimenteller Ausschaltung des Komplexes Subcommissuralorgan—Reissnerscher Faden. Untersuchungen am Rippenmolch (*Pleurodeles waltli* MICHAH. [1830]). Acta Zool. *52*, 23–68.
30. Hauser, R. (1972). Morphogenetic action of the subcommissural organ on tail regeneration in *Xenopus larvae*. Wilhelm Roux Arch. Entwickl. Mech. Org. *169*, 170–184.
31. Andrades, J.A., Becerra, J., and Fernández-Llebrez, P. (1994). Skeletal deformities of the gilthead sea bream (*Sparus aurata*, L.): study of the subcommissural organ (SCO) and Reissner's fiber (RF). Ann. Anat. *176*, 381–383.
32. Rodríguez, S., Vio, K., Wagner, C., Barría, M., Navarrete, E.H., Ramírez, V.D., Pérez-Fígares, J.M., and Rodríguez, E.M. (1999). Changes in the cerebrospinal-fluid monoamines in rats with an immunoneutralization of the subcommissural organ-Reissner's fiber complex by maternal delivery of antibodies. Exp. Brain Res. *128*, 278–290.
33. Vera, A., Recabal, A., Saldivia, N., Stanic, K., Torrejón, M., Montecinos, H., and Caprile, T. (2015). Interaction between SCO-spondin and low density lipoproteins from embryonic cerebrospinal fluid modulates their roles in early neurogenesis. Front. Neuroanat. *9*, 72.
34. Ségalen, M., Johnston, C.A., Martin, C.A., Dumortier, J.G., Prehoda, K.E., David, N.B., Doe, C.Q., and Bellaïche, Y. (2010). The Fz-Dsh planar cell polarity pathway induces oriented cell division via Mud/NuMA in *Drosophila* and zebrafish. Dev. Cell *19*, 740–752.
35. Schindelin, J., Arganda-Carreras, I., Frise, E., Kaynig, V., Longair, M., Pietzsch, T., Preibisch, S., Rueden, C., Saalfeld, S., Schmid, B., et al. (2012). Fiji: an open-source platform for biological-image analysis. Nat. Methods *9*, 676–682.
36. Haeussler, M., Schönig, K., Eckert, H., Eschstruth, A., Mianné, J., Renaud, J.B., Schneider-Maunoury, S., Shkumatava, A., Teboul, L., Kent, J., et al. (2016). Evaluation of off-target and on-target scoring algorithms and integration into the guide RNA selection tool CRISPOR. Genome Biol. *17*, 148.

37. Kimmel, C.B., Ballard, W.W., Kimmel, S.R., Ullmann, B., and Schilling, T.F. (1995). Stages of embryonic development of the zebrafish. Dev. Dyn. *203*, 253–310.

38. Jao, L.-E., Wente, S.R., and Chen, W. (2013). Efficient multiplex biallelic zebrafish genome editing using a CRISPR nuclease system. Proc. Natl. Acad. Sci. USA *110*, 13904–13909.

39. Lister, J.A., Robertson, C.P., Lepage, T., Johnson, S.L., and Raible, D.W. (1999). nacre encodes a zebrafish microphthalmia-related protein that regulates neural-crest-derived pigment cell fate. Development *126*, 3757–3767.

40. Thisse, C., and Thisse, B. (2008). High-resolution in situ hybridization to whole-mount zebrafish embryos. Nat. Protoc. *3*, 59–69.

41. Jaffe, K.M., Thiberge, S.Y., Bisher, M.E., and Burdine, R.D. (2010). Imaging Cilia in Zebrafish (Elsevier).

# STAR★METHODS

## KEY RESOURCES TABLE

| REAGENT or RESOURCE | SOURCE | IDENTIFIER |
|---|---|---|
| Antibodies | | |
| Anti-Acetylated tubulin, mouse monoclonal IgG2b (clone 6-11-B1) | Sigma Aldrich | Cat# T6793; RRID:AB_477585 |
| Anti-Reissner fiber, rabbit polyclonal | [5] | Courtesy of Dr. Stéphane Gobron |
| Anti-Gamma tubulin, mouse, monoclonal IgG1 | Sigma Aldrich | Cat# T5326; RRID:AB_532292 |
| Anti-GFP, chicken | Abcam | Cat #ab13970; RRID:AB_300798 |
| Anti-Phospho Histone 3 (PH3, Ser10), mouse | Cell Signaling Technology | Cat # 9706; RRID:AB_331748 |
| Donkey anti-rabbit IgG (H+L) Alexa Fluor-488 | Molecular Probes | Cat# A-21206; RRID:AB_141708 |
| Goat anti-mouse IgG (H+L) Alexa Fluor-568 | Molecular Probes | Cat# A-11004; RRID:AB_141371 |
| Donkey anti-mouse IgG Alexa Fluor-488 | Molecular probes | Cat# A-21202; RRID:AB_141607 |
| Goat anti-chicken IgG Alexa Fluor-488 | Molecular Probes | Cat# A11039; RRID:AB_142924 |
| Chemicals, Peptides, and Recombinant Proteins | | |
| FluoSpheresTM size kit #2, carboxylate-modified microspheres, yellow-green fluorescent (505/515), 2% solids, six sizes | Molecular Probes | F8888 |
| α-Bungarotoxin | TOCRIS | 2133 |
| Paraformaldehyde 16% SOL. EM Grade | Delta Microscopies | 15710 |
| *SphI* restriction enzyme from *Streptomyces phaeochromogenes* | Sigma Aldrich | 11026542001 |
| Experimental Models: Organisms/Strains | | |
| *hsc5Tg* (referred to as *Tg(β-actin:Arl13b-GFP)*) | [17] | ZFIN: ZDB-ALT-100721-1 |
| *scospondin$^{icm13}$* | This paper | N/A |
| *scospondin$^{icm15}$* | This paper | N/A |
| *traf3ip$^{tp49d}$* (referred to as *elipsa*) | [13] | ZFIN: ZDB-ALT-980413-466 |
| *cfap298$^{tm304}$* (referred to as *kurly*) | [14] | ZFIN: ZDB-ALT-980413-707 |
| *ift88$^{tz288b}$* (referred to as *oval*) | [14] | ZFIN: ZDB-ALT-980413-526 |
| *dzip$^{ts294e}$* (referred to as *iguana*) | [14] | ZFIN: ZDB-ALT-980203-1553 |
| Oligonucleotides | | |
| Forward PCR primer scospondin genotyping: GTGTCGGGGATTATTGCAAG | This paper | N/A |
| Forward PCR primer scospondin genotyping: TACTGGGTTACACCAACAGT | This paper | N/A |
| gRNA sequence for scospondin mutants generation: GGCTGGATGTGGAGCGCATG | This paper | N/A |
| Recombinant DNA | | |
| pCS2+ Ras-eGFP | [34] | N/A |
| Software and Algorithms | | |
| MATLAB and statistics toolbox release 2016b | The MathWorks | http://www.mathworks.com/ |
| R | The R project for statistical computing | https://cran.r-project.org/ |
| Fiji | [35] | https://Fiji.sc/ |
| CRISPOR | [36] | http://crispor.tefor.net/ |

## CONTACT FOR REAGENT AND RESOURCE SHARING

Further information and requests for resources and reagents should be directed to and will be fulfilled by the Lead Contact, Claire Wyart (claire.wyart@icm-institute.org).

## EXPERIMENTAL MODEL AND SUBJECT DETAILS

### Zebrafish

All procedures were performed on zebrafish embryos and larvae fore 5 days in accordance with the European Communities Council Directive (2010/63/EU) and French law (87/848) and approved by the Brain and Spinal Cord Institute (Institut du Cerveau et de la Moelle épinière, ICM). As experimentation on zebrafish larvae prior to 5 days old does not require approval of a protocol by the ethics committee, our project received the approval from the local ICM health and ethics committee. All experiments were performed on *Danio rerio* embryos of AB, Tüpfel long fin (TL) and *nacre* (*mitfa* homozygous mutant) background. Animals were raised at 28.5°C under a 14/10 light/dark cycle until the start of the experiment. The 24 hours post-fertilization (hpf) stage corresponds to 30-somite stage when raised at 28.5°C (according to [37]). Subsequent development of embryos was monitored from this stage until 48 hpf or later when needed.

## METHOD DETAILS

### scospondin mutants generation and genotyping

To generate loss of function alleles of the *scospondin* gene, we designed new guide RNA sequences using the CRISPOR tool (crispor.tefor.net/) [36]. To produce the selected sgRNA (GGCTGGATGTGGAGCGCATGcgg), we cloned two annealed oligos in the cloning vector, following previously published protocol [38]. We co-injected our synthetized sgRNA with an mRNA encoding the nls-zCas9-nls [38]. The efficiency of the sgRNA and further genotyping was evaluated from fin clip (heterozygous fish identification) or whole embryo DNA (genotype to phenotype correlation analysis, Figures S1D and S1E). Genomic DNA was isolated with proteinase K digestion in a lysis buffer overnight (10 mM Tris pH 8, 2 mM EDTA, 0.2% Triton X-100, 200 μg/mL Proteinase K). The *scospondin* mutations were genotyped by PCR using forward (GTGTCGGGGATTATTGCAAG) and reverse (TACTGGGTTACACCAACAGT) primers to generate a 500 bp product. Wild-type sequence was cleaved by *Sph1* to produce 280 and 220 bp bands, whereas the mutant bands were resistant to digestion. At later stages than 28 hpf, based on the strict correlation we observed between phenotype and genotype, we used the curled-down phenotype to identify homozygous mutants.

### scospondin mutants phenotype scoring

Embryos from *scospondin* $^{icm13/+}$ and *scospondin*$^{icm15/+}$ incrosses were manually dechorionated to assay body curvature defects (Figures 1C, S1B, and S1C) and scored at 30, 48 and 72 hpf. We used the angle (Θ) formed from the posterior part of the tail to the heart-yolk extension axis to classify the severity of the body axis curvature as follow: $\Theta \sim 180°$ for straight animals (score found for control siblings), $180° > \Theta > 90°$ classified as score 1, $\Theta \sim 90°$ classified as score 2 and $\Theta < 90°$ classified as score 3 in body axis curvature defect. To quantify the size of different body parts (Figure S1F; Table S1), anesthetized 48 hpf embryos from *nacre*$^{-/-}$ background [39] were laterally mounted in 1.5% low-melting point agarose and imaged with a Macroscope (Nikon AZ100M) equiped with a Digital Sight DS-Ri1 camera and a 2X AZ Plan Fluor objective (N.A. = 0.2). The resulting images were analyzed with Fiji [35] using the measure function and statistical analysis were performed using MATLAB. The height of the head was measured from the midbrain-hindbrain boundary to the intersection point between the head and the yolk. The tail length was measured from the otolith to the tip of the tail. The tail height was the mean of two measures corresponding to the two somites on both sides of the anus. Areas of the brain ventricles, eye and tail were traced according to the boundary of each structure with the surrounding tissues. Each measured parameter is exemplified in Figure S1F for a curled-down and straight sibling and is reported in Table S1 for both *scospondin* alleles. To analyze morphometric parameters at the level of the trunk, 60 pg of Ras-eGFP mRNA [34] were injected into one cell stage embryos from *scospondin*$^{icm13/+}$ incrosses. Embryos were immunostained against GFP at 30 hpf (see Immunohistochemistry and Fixed Tissue Imaging) and used to quantify the height of the spinal cord, floor plate and notochord (exemplified in Figure S1G, and reported in Table S1). A Z projection of a 3 μm stack was performed in a 100 μm wide and 15 μm height region and located between segments 10 to 14 of the trunk for each analyzed embryo.

### Immunohistochemistry and fixed tissue imaging

Embryos were chemically dechorionated using Pronase incubation in Danieau buffer for 10 min at 28.5°C as previously described [40] and euthanized in 0.2% MS 222 (Sigma) prior to fixation. Embryos from 24 to 40 hpf were fixed 4 hr to overnight in 4% paraformaldehyde (PFA, Delta Microscopies) at 4°C. Larvae at 48 and 72 hpf were fixed 2 hr in 4% PFA and 3% sucrose at 4°C, and skin from the rostral trunk was partially removed and yolk was removed. Samples from 24 to 40 hpf embryos were blocked over-night in a solution containing 0.5% Triton, 1% DMSO, 10% normal goat serum and 2 mg/mL BSA. Samples from 48 to 72 hpf larvae were blocked in 0.7% Triton, 1% DMSO, 10% NGS and 2 mg/mL BSA. Primary antibodies were incubated one to two nights at 4°C in a buffer containing 0.5% Triton, 1% DMSO, 1% NGS and 1 mg/mL BSA. All secondary antibodies were from Molecular Probes, used at 1:500 in blocking buffer, and incubated 2.5 hr at room temperature. The following primary antibodies were used for *in toto* immunohistochemistry: rabbit anti-Reissner fiber (polyclonal, custom-made, 1:200) [5], mouse anti-Acetylated-tubulin (monoclonal, Sigma T6793, 1:500), mouse anti-Gamma-tubulin (monoclonal, Sigma, T5326, 1:200), mouse anti-PH3 (Ser10, Cell Signaling Technology 9706, 1:250), and chicken anti-GFP (Abcam, ab13970, 1:500). The following secondary antibodies were used (at 1:500): Alexa Fluor-488 donkey anti-rabbit IgG A21206, Alexa Fluor-488 donkey anti-mouse IgG A21202, Alexa Fluor-568 goat anti-mouse A11004, Alexa Fluor-488 goat anti chicken IgG A11039. Anti-Reissner fiber antibody produced background labeling in the skin. Systematic omission

of the primary antibody confirmed the specificity of the results from immunostaining. Whole mount zebrafish embryos (dorsal or lateral mounting in Vectashield Antifade Mounting Medium) were imaged on an Olympus FV-1000 or FV-1200 confocal microscope equipped with a 40X water immersion objective (imaging of brain secretory structures: SCO, 1 μm optical section, N.A. = 0.8), or 40X oil immersion objective (imaging of the trunk and spinal cord, 0.5 μm optical section, N.A. = 1.3). Images were then processed using Fiji [35].

## Quantification of apparent cilia length and density

Apparent cilia length quantification was performed as previously described [41]. A Z projection of a 3 μm stack was performed on regions of the spinal cord allocated to a single segment, and isolated to measure apparent cilia length. Spinal cord regions located between segments 10 to 14 were imaged at different developmental time points for control siblings, *scospondin*$^{icm13/icm13}$ (Figure S2), and *scospondin*$^{icm15/icm15}$ mutants (*data not shown*). Ciliated structures projecting in the lumen of the central canal were then manually traced using Fiji (https://Fiji.sc/) [35]. Cilia density was quantified based on Gamma-tubulin immunostaining. Briefly, a 3 μm stack Z projection was performed in a 100 μm wide and 15 μm height region surrounding the central canal and located between segments 10 to 14 of the trunk. After background substraction, objects corresponding to basal bodies were identified using the 2D/3D Object Counter plugin in Fiji that was used to locate centers of mass maps for segmented objects shown in Figure S2C.

## Live imaging of spinal cord cilia

30 hpf embryos from *Tg(β-actin:Arl13b-GFP; scospondin*$^{icm13/+)}$ incrosses were manually dechorionated, laterally mounted in 1.5% low-melting point agarose, and paralyzed by injecting 1-2 nL of 500 μM alpha-bungarotoxin (TOCRIS) in the caudal muscles of the trunk. A spinning disc confocal microscope (Intelligent Imaging Systems, Denver) equipped with a 63X water immersion objective (N.A. = 1) was used to focus on a single optical section (0.5 μm) of the central canal, anatomically positioned between segments 10 to 14 of control siblings and curled down *scospondin*$^{icm13/icm13}$ embryos. Images were acquired at 17 Hz for 28 s to generate a recording of cilia motility.

## Fluorescent beads injection in the CSF

30 hpf embryos were manually dechorionated, mounted in 1.5% low-melting point agarose, and paralyzed by injecting 1-2 nL of 500 μM alpha-bungarotoxin (TOCRIS) in the caudal muscles of the trunk. 20 nm carboxylate FluoSpheres emitting at 505/515 nm (yellow/green, F8888, Molecular Probes) were diluted to a 2% concentration in artificial CSF (containing in mM: 134 NaCl, 2.9 KCl, 1.2 MgCl2, 10 HEPES, 10 glucose, 2 CaCl2; 290 mOsM ± 3 mOsm, pH adjusted to 7.8 with NaOH), and then sonicated for 2-3 s. The injection needle was inserted through the roof plate of the hindbrain ventricle, and a volume of 1 to 3 nL was injected using a Picospitzer device (World Precision Instruments). Ventricle injection quality was evaluated based on hindbrain ventricle filing with the fluorescent beads. Embryos displaying damages in the epithelia of the ventricle walls were discarded before the fluorescent imaging step.

## Beads transport analysis

To measure CSF transport, groups of 3 to 5 embryos at 30 hpf were injected simultaneously and beads front progression was monitored over time using an epifluorescence microscope (Zeiss AX10 Imager) equipped with a 40X water immersion objective (N.A. = 1.0) and HXP 120C illumination lamp. The progression of the fluorescent beads front transported along the central canal was assessed using a GFP/YFP filter (500/25 bandpass excitation filter, 515 nm dichroic and 535/30 nm bandpass emission filter). Transmitted light signals allowed detecting segments limits that were used as anatomic landmarks to assess the progression of the fluorescence signal. Representative images shown in Figures 3 and S3 were acquired on an Apotome2 microscope equipped with an Axiocam camera and a Zeiss W N-Achroplan 10X objective (N.A. = 0.3), using a HXP 120C illumination lamp.

## Fluorescent beads tracking

For CSF flow experiments, time-lapse images were acquired at 26°C using a thermostatic chamber mounted on an inverted Leica DMI8 spinning disk confocal microscope equipped with a Hamamatsu Orca Flash 4.0 camera, using a 40X water immersion objective (N.A. = 0.8, pixel size 188 nm). Images from segment 10 to 14 of the trunk were acquired at a frame rate of 10 Hz for 30 s using Metamorph software (http://www.moleculardevices.com). The beads behavior was analyzed in Fiji [35]. First, time lapses were rotated and cropped to isolate a portion of the central canal. Stacked images were then re-sliced (Figure 3A) and a maximum Z projection of a stack over 1.5 to 1.9 μm was performed on dorsal and ventral regions of the central canal to obtain kymographs (Figure 3B). Trajectories of individual beads were then manually traced (Figure 3B) using a custom MATLAB script used to calculate velocities along the rostro-caudal axis from trajectories start and end points.

## Analysis of Reissner fiber formation defects

To quantify the distribution of the Reissner fiber material along the rostro-caudal axis of the central canal, 48 hpf control sibling and *iguana*, *oval*, *elipsa* and *kurly* embryos (and 24 hpf for *elipsa* and *kurly*) were immunolabelled against the Reissner fiber and imaged using an epifluorescence microscope (Zeiss AX10 Imager) equipped with a 40C water immersion objective (N.A. = 1.0) and HXP 120C illumination lamp. At 24 hpf, mutants with impaired cilia were genotyped as previously described [13, 22]; at later stages, we used the curled-down phenotype to identify homozygous mutants. Six categories of Reissner fiber aggregation states were qualitatively

defined (Figure 2B). The rostral and caudal limit of the distribution of each category was assessed in each fish using a GFP/YFP filter (500/25 bandpass excitation filter, 515 nm dichroic and 535/30 nm bandpass emission). Transmitted light signals allowed detecting segments limits that were used as anatomic landmarks. Distributions of Reissner fiber aggregation defects (Figures 4C and 4E) were represented for each category as the mean value ± SEM of the segment number reached at rostral and caudal limits. Representative images (Figures 4A, 4B, and 4D) were acquired on a FV-100 confocal microscope.

## QUANTIFICATION AND STATISTICAL ANALYSIS

All values are represented as boxplots (median ± interquartile range) or mean ± SEM (stated for each in the figure legend). All statistics were performed using R (https://cran.r-project.org/) MATLAB and Excel. Statistical details related to sample size, p values, t-values and degrees of freedom (dF) are reported in the figure legends. In figure panels, asterisks denote the statistical significance calculated by two-tailed t test for samples with unequal variance (Welch t test): *, $p < 0.05$; **, $p < 0.01$; ***, $p < 0.001$; ns, $p > 0.05$.

Current Biology, Volume *28*

# Supplemental Information

## The Reissner Fiber in the Cerebrospinal Fluid

## Controls Morphogenesis of the Body Axis

**Yasmine Cantaut-Belarif, Jenna R. Sternberg, Olivier Thouvenin, Claire Wyart, and Pierre-Luc Bardet**

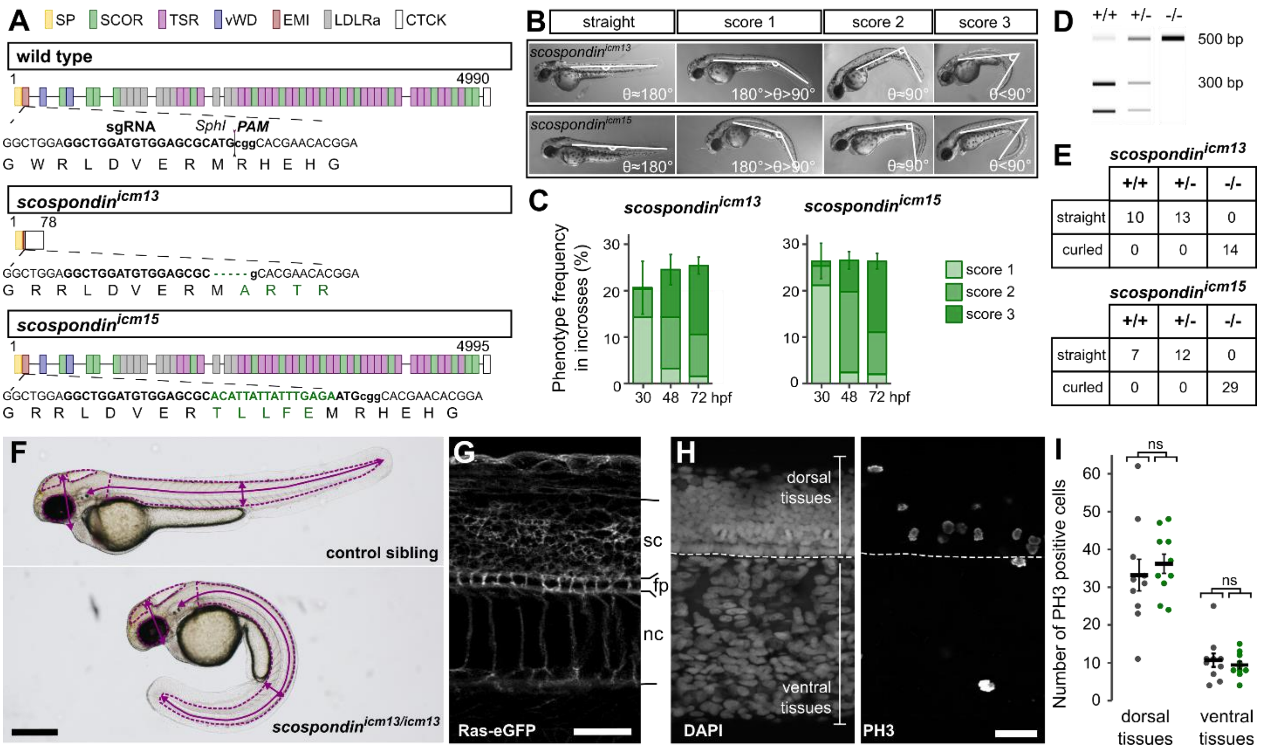


**Figure S1. Embryonic posterior axis defects strictly correlate with *scospondin* mutated alleles, *related to Figure 1 and Table S1***

**A.** CRISPR/Cas9-mediated genome editing leading to a nonsense mutation, and a five amino acids insertion in the EMI domain of SCO-spondin for the *scospondin*$^{icm13}$ and *scospondin*$^{icm15}$ allele, respectively (SP: signal peptide, EMI: Emilin domain, SCOR: SCO-spondin repeats, vWD: von Willebrand D domain, LDLRa: Low-Density Lipoprotein Receptor type A Repeat, TSR: Thrombospondin type 1 Repeats, CTCK: C-terminal Cystine Knot, see [S1]). **B.** *scospondin* mutants show different levels of severity in body axis curvature at 48 hpf (score 1, 2, 3). Curvature defects are classified according to the angle (θ) between the tail and the rostral axis. **C.** Around 25% of embryos showed curvature defects from 30 hpf onwards, in agreement with Mendelian distributions. Curvature defects (%, mean ± SEM) were more pronounced over time for both *scospondin* alleles (from 30 to 72 hpf, n=386 and 248 embryos for *icm13* and *icm15* alleles respectively, n= 3 clutches). **D.** Embryos from *scospondin*$^{icm13/+}$ and *scospondin*$^{icm15/+}$ incrosses were genotyped at 72 hpf using the *Sph1* restriction site loss in *scospondin* mutants. **E.** All *scospondin* homozygous mutants showed the curled-down phenotype and it was observed neither in the *scospondin* heterozygous nor in wild type embryos. The same is true for trans-heterozygous *scospondin*$^{icm13/icm15}$ embryos (*data not shown*). **F.** To perform  morphometric analysis of *scospondin* mutants, 48 hpf control and curled-down embryos from both alleles were measured for the eye, brain ventricles and tail area (dotted lines) as well as head and trunk height and tail length (solid lines). (Scale bar represents 1 mm.) **G.** To perform morphometric measurements at the level

of the trunk, 30 hpf control and *scospondin*$^{icm13/icm13}$ embryos injected at one cell stage with the Ras-eGFP mRNA were immunostained against GFP. The membrane-tag fluorescence was used to measure the spinal cord (sc), floor plate (fp) and notochord (nc) height in both straight and curled-down siblings. **H.** Representative images of a 30 hpf control embryo immunostained against phospho-histone 3 (PH3, right) allowing to detect cell proliferation. DAPI counterstaining of the nuclei (left) allowed differentiating dorsal and ventral tissues above and under the floorplate (dotted line) respectively. **I.** Quantification of PH3 positive cells in control (black) and *scospondin*$^{icm13/icm13}$ (green) embryos at 30 hpf in dorsal and ventral tissues. n=10 embryos for each condition. p= 0.57; 0.55, t= -0.58; 0.61 and df= 15; 14 for dorsal; ventral tissues respectively; two-tailed t-test. Scale bars represent 30 µm in **G** and **H**.

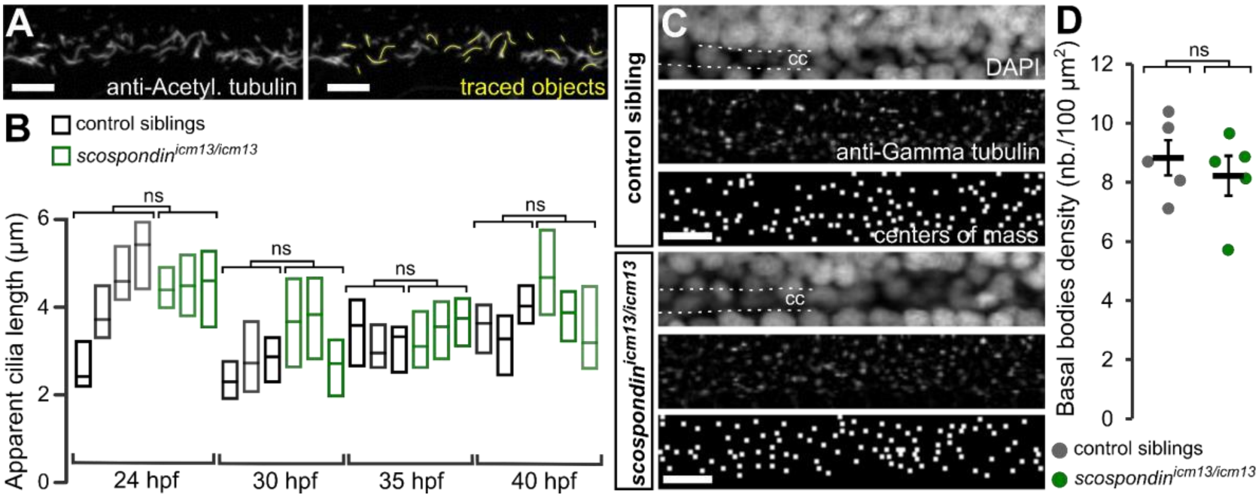


**Figure S2. Cilia length and density are unchanged in *scospondin*$^{icm13/icm13}$ mutants, *related to Figure 2 and Video S1***

**A**. Z projection of a stack of lateral optical sections (depth= 3 µm) of the spinal cord of a 30 hpf embryo immunostained against Acetylated-tubulin (left) and after tracing to estimate cilia length (yellow). Scale bar represents 10 µm. **B**. Cilia length is similar in *scospondin*$^{icm13/icm13}$ embryos (green) compared to control siblings (black). (Median ± interquartile range, n= 83; 105; 95 and 95 and 67; 96; 94 and 89 cilia at 24; 30; 35 and 40 hpf for control and *scospondin*$^{icm13/icm13}$; p = 0.53; 0.14; 0.53 and 0.61, respectively. t= -0.7; -1.8; -0.67 and -0.55, respectively; df = 3.05; 3.4; 3.9 and 2.9, respectively, two-tailed t-test). Each box plot represents a single fish; color intensity reflects the number of measured objects for each fish. Similar results were obtained for *scospondin*$^{icm15/icm15}$ embryos (*data not shown*). **C, D.** Z projection of stacks of lateral optical sections (depth = 3 µm) of the spinal cord stained with DAPI and against Gamma-tubulin in a 30 hpf control sibling (top) and *scospondin*$^{icm13/icm13}$ curled-down embryo (bottom), allowing to detect basal bodies around the central canal (cc). Centers of mass of detected objects were used to quantify cilia density shown in **D** as the number of basal bodies per 100 µm$^2$. n= 5 control (black) and 5 *scospondin*$^{icm13/icm13}$ embryos (green). p= 0.52, t= 0.68, df= 8, two-tailed t-test. Each point corresponds to a single fish**.** Scale bars represent 10 µm in **C**.

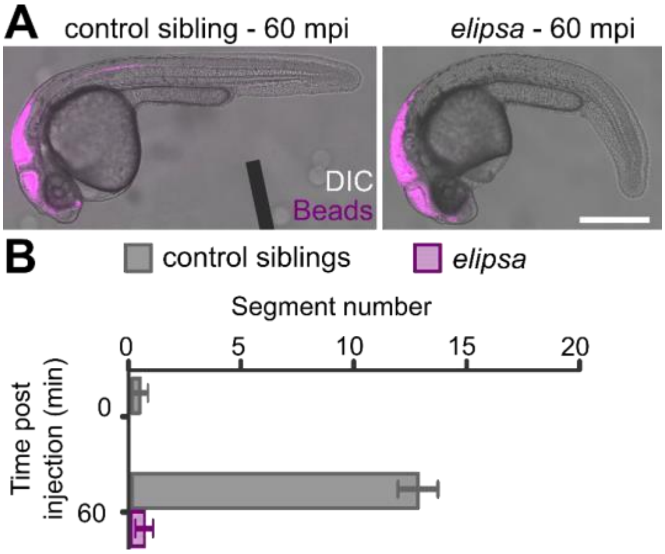


**Figure S3. Transport of exogenous fluorescent beads in the cerebrospinal fluid is abolished in *elipsa* mutants with defective cilia, *related to Figure 3 and Video S2*.**

**A.** Superimposed images of transmitted light (DIC) and fluorescent 20-nm diameter beads (magenta) injected in the third ventricle of 30 hpf control sibling and *elipsa* mutant embryos. Beads are transported down the central canal in straight control siblings, but not in *elipsa* mutants as shown 60 minutes post-injection (mpi). Scale bar represents 0.5 mm. The progression of the fluorescence front in the central canal is quantified in **B** as the segment number reached 60 minutes after injection (mean ± SEM) in control sibling (n = 3) and *elipsa* mutant embryos (n = 5) (p = 0.0014; t = 12.46, df = 2.84, two tailed t-test).

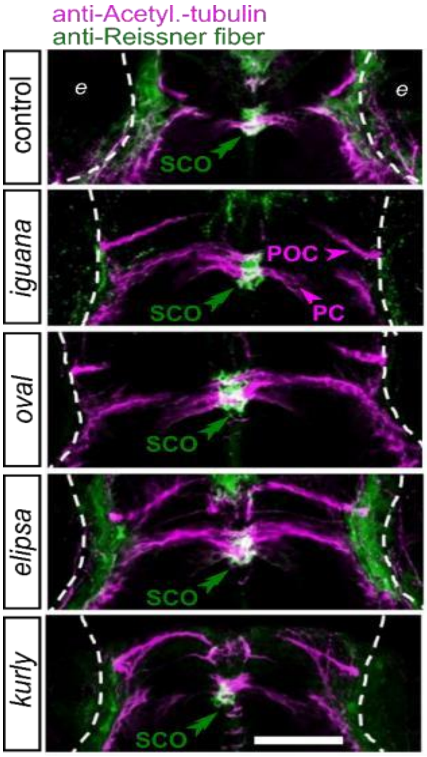


**Figure S4. The sub-commissural organ is immunoreactive for the Reissner fiber material in mutants with defective cilia, *related to Figure 4*.**

Z projection of stacks of dorsal optical sections (depth = 23 - 26 µm) of 48 hpf embryos immunostained against Acetylated-tubulin (magenta) allowing to detect axonal tracts (POC: post-optic commissure, PC: posterior commissure, arrowheads), and the Reissner fiber material (green) in the sub-commissural organ (SCO, double arrowheads). Control embryos (here an *iguana* sibling) as well as *iguana*, *oval*, *elipsa* and *kurly* mutants show immunoreactivity for the Reissner fiber material in the SCO. Rostral, top. e: eye. Scale bar represents 50 µm.

| | allele | straight siblings | curled-down embryos | t-value | df | *p-value* |
|---|---|---|---|---|---|---|
| **eye area (µm$^2$)** | ***icm13*** | **55 368** +/- 2 532 | **53 777** +/- 2 492 | 0.878 | 22.0 | *0.390* |
| | ***icm15*** | **59 843** +/- 2 342 | **56 828** +/- 2 432 | 1.751 | 20.8 | *0.095* |
| **anterior ventral area (µm$^2$)** | ***icm13*** | **12 123** +/- 866 | **12 859** +/- 1 023 | -1.076 | 21.4 | *0.294* |
| | ***icm15*** | **12 995** +/- 677 | **12 216** +/- 715 | 1.551 | 20.8 | *0.136* |
| **posterior ventral area (µm$^2$)** | ***icm13*** | **12 083** +/- 828 | **14 009** +/- 2 090 | -1.679 | 14.4 | *0.115* |
| | ***icm15*** | **10 745** +/- 1085 | **11 347** +/- 1 981 | -0.522 | 15.6 | *0.609* |
| **head height (µm)** | ***icm13*** | **449.9** +/- 10.0 | **450.4** +/- 10.7 | -0.062 | 21.9 | *0.951* |
| | ***icm15*** | **452.3** +/- 8.6 | **444.3** +/- 8.3 | 1.317 | 21.0 | *0.202* |
| **tail area (µm$^2$)** | ***icm13*** | **427** +/- 21 **.10$^3$** | **409** +/- 14 **.10$^3$** | 1.405 | 18.9 | *0.176* |
| | ***icm15*** | **480** +/- 16 **.10$^3$** | **435** +/- 24 **.10$^3$** | 3.045 | 17.3 | *0.007* |
| **tail length (µm)** | ***icm13*** | **2 618** +/- 49 | **2 695** +/- 45 | -2.297 | 21.8 | *0.032* |
| | ***icm15*** | **2 741** +/- 68 | **2 660** +/- 206 | 0.727 | 12.2 | *0.481* |
| **tail height (µm)** | ***icm13*** | **206.3** +/- 7.3 | **196.0** +/- 4.4 | 2.373 | 18.1 | *0.029* |
| | ***icm15*** | **217.0** +/- 6.3 | **204.4** +/- 4.7 | 3.158 | 19.9 | *0.009* |
| **spinal cord height (µm)** | ***icm13*** | **27.08** +/- 1.84 | **26.39** +/- 3.73 | 0.329 | 5.8 | *0.754* |
| **floor plate (µm)** | ***icm13*** | **3.79** +/- 0.55 | **3.87** +/- 0.37 | -0.242 | 7.0 | *0.816* |
| **notochord (µm)** | ***icm13*** | **27.18** +/-3.23 | **28.45** +/- 1.75 | -0.679 | 6.2 | *0.522* |

**Table S1. Morphometric analysis of *scospondin* mutant embryos, *related to Figure 1 and Figure S1***

We measured the size of different body parts of 48 hpf embryos, as depicted in Figure S1F. Between 5 and 6 embryos of each phenotype (curled-down mutants or straight siblings) from three independent clutches were measured for both alleles and displayed as means +/- confidence interval. We observed no signs of microcephaly (eye size and head height), hydrocephaly (head height, ventricle areas) or shortening of the body (tail length and area). Note that all p-values are above the significance threshold after Bonferroni correction (p< 0.007). We also performed measurement on confocal images of *scospondin*$^{icm13/icm13}$ and control siblings expressing a membrane-tethered GFP at 30 hpf (see Figure S1G). No major changes in the height of the spinal cord, the floor plate and the notochord were observed.

**Supplemental References**

**S1**. Meiniel, O., and Meiniel, A. (2007). The complex multidomain organization of SCO-spondin protein is highly conserved in mammals. Brain Res. Rev. 53, 321–327.
